# The Transcriptional Mediator Component Med12 Is Required for Hindbrain Boundary Formation

**DOI:** 10.1371/journal.pone.0019076

**Published:** 2011-04-21

**Authors:** Sung-Kook Hong, Igor B. Dawid

**Affiliations:** 1 Laboratory of Molecular Genetics, Program in Genomics of Differentiation, Eunice Kennedy Shriver National Institute of Child and Human Development, National Institutes of Health, Bethesda, Maryland, United States of America; 2 Molecular Genetics Branch, National Human Genome Research Institute, National Institutes of Health, Bethesda, Maryland, United States of America; Texas A&M University, United States of America

## Abstract

**Background:**

Rhombomere boundaries form during hindbrain segmentation and are critical for maintaining segmental integrity and regulating migration in the hindbrain. Some genetic models affecting hindbrain boundary formation have been described, but involvement of components of the transcriptional mediator complex in boundary formation has not reported so far.

**Principal Findings:**

The *kto/med12* mutant zebrafish, which affects the Mediator component Med12, causes specific loss of rhombomere boundary cells even though segmentation of the hindbrain takes place at least in part. In *kto* mutant embryos, cells forming rhombomere boundaries were largely absent as indicated by the use of several marker genes. While no obvious increase in cell death was observed, we found a notable reduction of cell proliferation in the hindbrain of *kto* mutant zebrafish.

**Conclusions:**

The *kto/med12* mutation results in specific defects of boundary cell formation in the zebrafish hindbrain.

## Introduction

The Mediator is an evolutionarily conserved multi-subunit complex that functions to bridge regulatory regions to the RNA polymerase II initiation complex in eukaryotic cells [1], [2], [3], [4], [5]. We have reported isolation of a zebrafish mutant named to *kto* that encodes Mediator component Med12, also called Trap230 [6]. *Kto/med12* mutants show multiple phenotypes, including defects in brain, neural crest, and kidney development. Other investigators have reported that the zebrafish *med12* gene functions in neuronal and endoderm development, and that Med12 acts as a co-activator for Sox9 [7], [8], [9]. In the mouse, a hypomorphic mutation of *Med12* leads to neural tube closure and other defects, and the null mutant is lethal at about E7.5 [10]. In humans the *MED12* gene is associated with X-linked disorders characterized by mental retardation [11], [12], [13].

In studying the phenotype of the *kto/med12* mutation we focused our attention on the hindbrain in the zebrafish embryo. During vertebrate development the hindbrain is segmented into units named rhombomeres. Many aspects of hindbrain differentiation and organization depend on this segmental order [14], and migration of neural crest cells follows rhombomere patterns [15]. During segmentation the lineage of hindbrain cells is restricted to rhombomere compartments, and no mixing between adjacent segments takes place [16]. Furthermore, even and odd-numbered rhombomeres show distinct affinities with apparent alternating properties [17]. Several transcription factors, including *kreisler/valentino/mafba* [18], [19], [20], [21], *egr2b/krox-20* [22], and certain Hox group proteins [14] are expressed in rhombomeric patterns and play a role in hindbrain segmentation.

Rhombomere organization depends critically of several signaling pathways. The receptor tyrosine kinase EphA4 and its ligand ephrinB2 are expressed in an alternating rhombomeric pattern and have an essential role in segment-specific cell sorting during hindbrain segmentation [23], [24], [25], [26]. Notch-delta signaling likewise is crucial for maintaining boundary structure in the embryonic hindbrain [27], [28]. Differentiation and maintenance of boundary and non-boundary regions of rhombomeres is regulated by the close interaction of Wnt and Notch signaling. Notch and Wnt1 are expressed in boundary cells whereas the Notch ligand Delta is expressed in adjacent stripes also named paraboundary domains, and Notch activity in the boundaries is enhanced by the modifier Radical Fringe, a glycosyltransferase [27], [29], [30], [31]. The appropriate expression and activity of these factors in the boundary and adjacent regions is required to maintain the normal rhombomeric organization of the hindbrain.

In the present study we found that hindbrain boundaries do not form in *kto/med12* mutant embryos, as visualized by the use of several marker genes. Nevertheless, rhombomeric organization does arise in the mutant hindbrain and some rhombomere markers are expressed appropriately while others are reduced or lost. It appears that expression of markers for odd rhombomeres is lost more generally that those for even rhombomeres. Further we find that cell proliferation is reduced in the hindbrain of mutant embryos, whereas cell death appears unaffected. This study demonstrates the specific requirement of a Mediator component for the appropriate organization of hindbrain segments in the zebrafish.

## Results

### Hindbrain phenotype in the *kto/med12* mutant

**Figure 1:**
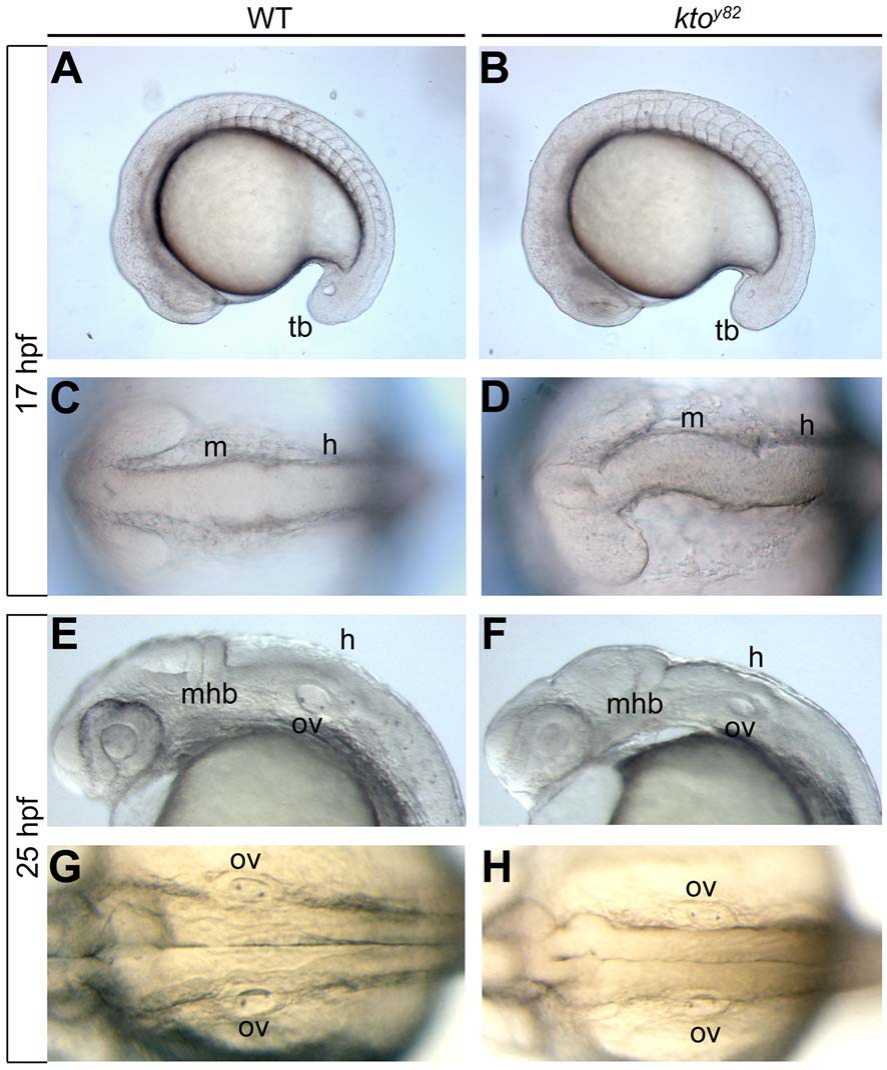
The hindbrain phenotype of *kto^y82^*. Live images of lateral (A,B,E,F) and dorsal views (C, D, G, H) of the developing hindbrain at 17 hpf (A–D) and 25 hpf (E–H). The early midbrain and hindbrain regions are malformed at 17 hpf in mutant embryos (D), and no ventricle is visible at 25 hpf (H). h, hindbrain; m, midbrain; mhb, mid-hindbrain boundary; ov, otic vesicle; tb, tail bud.

We have reported previously that the *kto* mutant, which encodes the Med12 Mediator component, shows malformed brain structures including defective ventricle inflation in the fore- and midbrain and incomplete ventricle formation in the hindbrain [6]. Here we focus more closely on the hindbrain from the beginning stages of its segmentation. The earliest stage at which boundary formation in zebrafish can be observed is the 5 somite stage (11.7 hpf) [32], and by mid-somitogenesis (17 hpf) the segmental organization of the hindbrain, and morphologically visible boundaries are established. At this stage, defects are observed in brain development of *kto* mutant embryos, including a severely distorted midbrain (Figure 1A–D). At 24 hpf brain structure has become further disorganized, including defects in the hindbrain such as failure to form a normal-sized ventricle and a poorly defined separation along the dorsal midline (Figure 1E–H). The reduced size of fore-, mid-, and hindbrain but largely unaffected size of the trunk has been reported previously [6].

### Med12 is not required for the initiation of hindbrain segmentation

**Figure 2:**
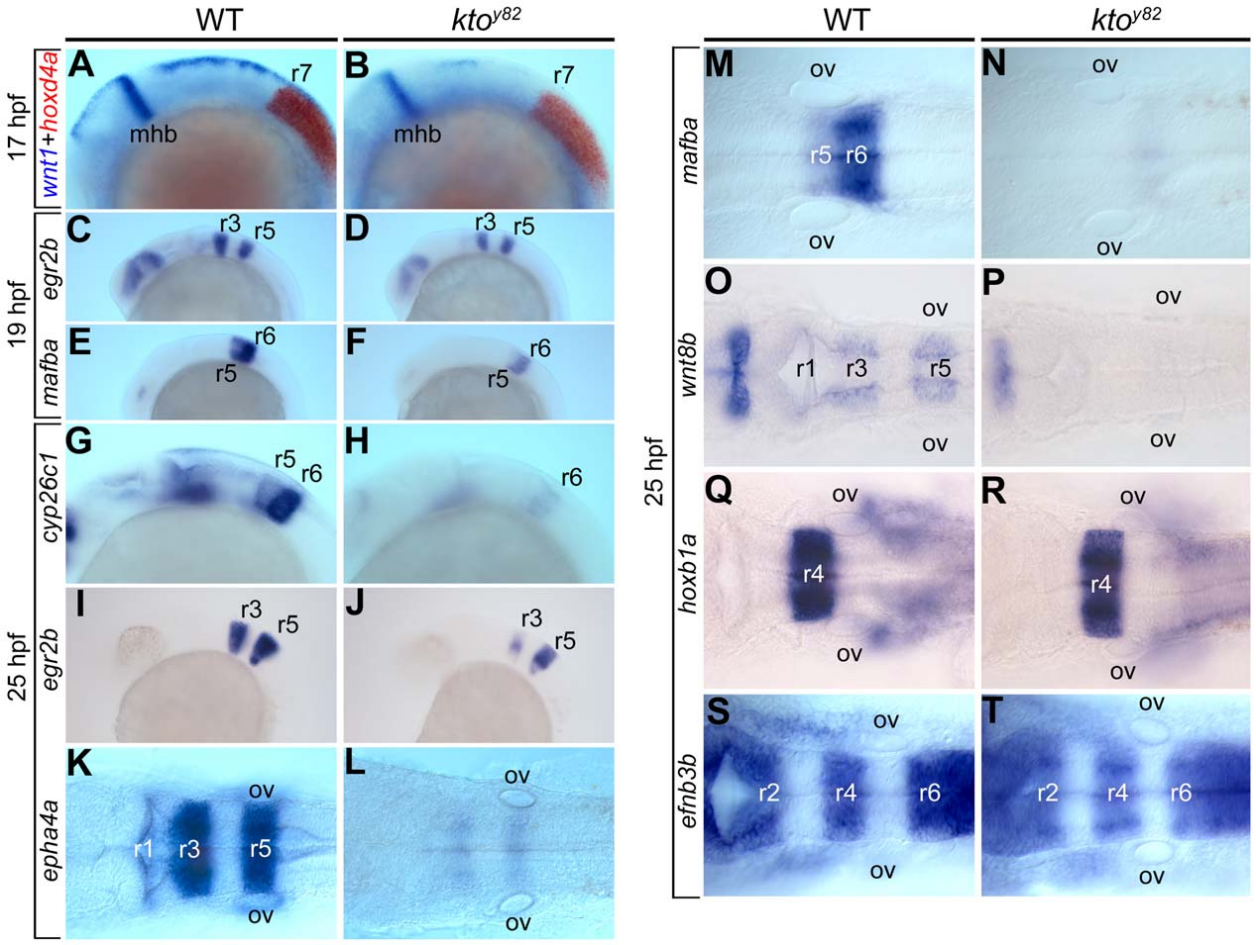
Hindbrain segmentation in the *kto^y82^* mutant. Lateral (A–J) and dorsal views (K–T) of wt and mutant embryos, as indicated, stained by whole mount in situ hybridization. Two-color in situ hybridization with *wnt1* (blue) and *hoxd4a* (red) at 17 hpf (A,B). Expression of *egr2b* (C,D) and *mafba* (E,F) at 19 hpf, and *cyp26c1*, *egr2b*, *epha4a*, *mafba*, *wnt8b*, *hoxb1a* and *efnb3b* at 25 hpf (G–T). mhb, mid-hindbrain boundary; ov, otic vesicle; r, rhombomere.

To examine hindbrain segmentation in more detail, we performed in situ hybridization with various segmental markers during different stages of development in wild type (wt) and mutant embryos. We first wished to visualize the entire hindbrain in mid-somitogenesis stage wt and *kto* embryos, using two-color in situ hybridization with the markers *wnt1* and *hoxd4a* [21], [33] that span the region from the mid-hindbrain boundary to rhombomere7 (r7). *Wnt1* expression at the dorsal midline was significantly reduced but was maintained at a normal level at the mid-hindbrain boundary, and *hoxd4a* in rhombomere7 was expressed normally in the mutant embryos (Figure 2A,B). Slightly later at 19 hpf, two transcription factors known to function in hindbrain segmentation, *egr2b/krox-20* (specific for r3 and r5) and *mafba/valentino* (specific for r5 and r6) show somewhat reduced expression within their normal domains (Figure 2C–F). By 25 hpf, multiple markers are available to assess the development of individual rhombomeres within the hindbrain, including *epha4a* and *wnt1* that mark r1, r3, r5 [33], [34], *efnb3b* marking r2, r4, r6 [23], *hoxb1a* for r4 [35], and *cyp26c1* and *mafba* for r5, r6 [20], [21], [36]. Experiments using these markers showed that expression of *egr2b, epha4*, and *wnt8b* in odd-numbered rhombomeres was strongly reduced or totally lost in *kto* mutant embryos, as was the weak expression of *cyp26c1* and *mafba* in r5 (Figure 2G–P). In contrast, the behavior of even-numbered rhombomeres varied: expression of *cyp26c1* and *mafba* in r6 was lost whereas *hoxb1a* in r4 and *efnb3b* in r2, r4, r6 were expressed at normal levels in the mutant embryos (Figure 2G,H,M,N,Q–T). These observations resist rationalization in a simple way that interprets the effects as characteristic for even or odd-numbered segments. However it may be noted that *cyp26c1* and *mafba* whose expression in r6 is lost in the mutant, are also expressed in r5 and therefore may not be suitable odd/even rhombomere markers. If these two markers are set aside we find that all genes expressed in odd-numbered rhombomeres are extinguished while those in even-numbered rhombomeres are unaffected by the *kto* mutation. In any case the data suggest a complex pattern of regulation of gene expression in the hindbrain segments, only some of which depend on the function of Med12.

### Loss of hindbrain boundary cells and neurons

**Figure 3:**
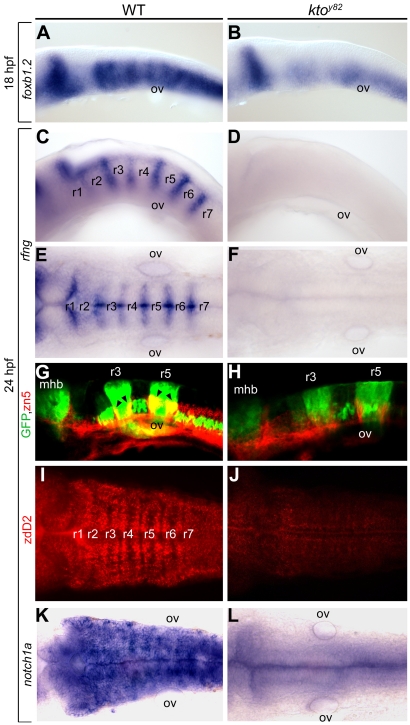
Specific loss of hindbrain boundaries in *kto^y82^* mutants. Lateral (A–D,G,H) and dorsal views (E,F,I–L) of wt (A,C,E,G,I,K) and *kto^y82^* mutant embryos (B,D,F,H,J,L) at 18 hpf (A,B) and 24 hpf (C–L). (A–F, K,L) in situ hybridization. (A,B) Expression of the earliest hindbrain boundary marker *foxb1.2*. (C–F) Completely loss of *rfng* expression in hindbrain boundaries. (G–J) Confocal images of hindbrain boundary neurons; immunostaining with zn5 (red) in pGFP-5.3 transgenic zebrafish (green) (G,H); staining with zebrafish delta D antibody, zdD2 (I,J). (K,L) *notch1a* expression in the hindbrain. mhb, mid-hindbrain boundary; ov, otic vesicle; r, rhombomere.

Morphologically visible boundaries form between rhombomeres during hindbrain development, and boundary formation may depend on the distinct properties of successive odd and even-numbered segments [37]. Based on our finding that marker gene expression is dramatically reduced in odd-numbered segments and variably retained in even-numbered segments we asked whether rhombomere boundaries are formed in *kto* mutant embryos. The first rhombomere boundaries appear at early somite stages and are well developed by 17–18 hpf, when they can be detected using *foxb1.2* as a marker [19]. We found that *foxb1.2* expression was substantially reduced in the hindbrain of mutant embryos (Figure 3A,B). The effect was even more pronounced at 24 hpf as visualized by *rfng* [27], [29], [30], which was entirely lost from the hindbrain of *kto* mutant embryos (Figure 3C–F). It is known that rhombomere organization is important for the survival of neurons in the hindbrain. To visualize rhombomeres in live embryos we have generated homozygous *kto^y82^* mutant embryos in the pGFP5.3 transgenic line that expresses GFP in rhombomeres 3 and 5 [38]. Whole mount immunostaining with zn-5 antibody (detecting DM-GRASP) was used to label commissural axons juxtaposed to segmental boundaries [39]. Within r3 and r5, these axons were visualized in yellow as a result of overlap of GFP and antibody staining (Figure 3G). In mutant embryos, GFP-expression in r3 and r5 was maintained at a reduced level, but zn-5 positive axons were largely abolished (Figure 3H). Thus, axons close to the rhombomere boundaries were lost in the *kto* mutant. The Notch-Delta signaling pathway has a critical role in segmentation and in neuronal differentiation in the hindbrain. Whole mount immunostaining using zdD2 antibody visualizes deltaD in para-boundary regions of the zebrafish hindbrain [40], and this signal was lost entirely in the *kto* mutant (Figure 3I,J). Likewise expression of the receptor *notch1a* in the hindbrain [41] was abolished in the mutant embryos (Figure 3K,L). Thus, signaling pathways essential for hindbrain segmentation are severely impaired in *kto/med12* mutant embryos.

The results described above for mutant zebrafish were confirmed by using wild type fish injected with Med12 antisense morpholino (MO). In addition to *rfgn, foxb1.2,* and *notch1a* we also found the loss of expression of *wnt1* in the rhombomere boundary regions (Figure S1). Note that *wnt1* expression in the midbrain-hindbrain boundary is maintained albeit it at a reduced level.

### Reduced proliferation in *kto/med12* mutant hindbrain

**Figure 4:**
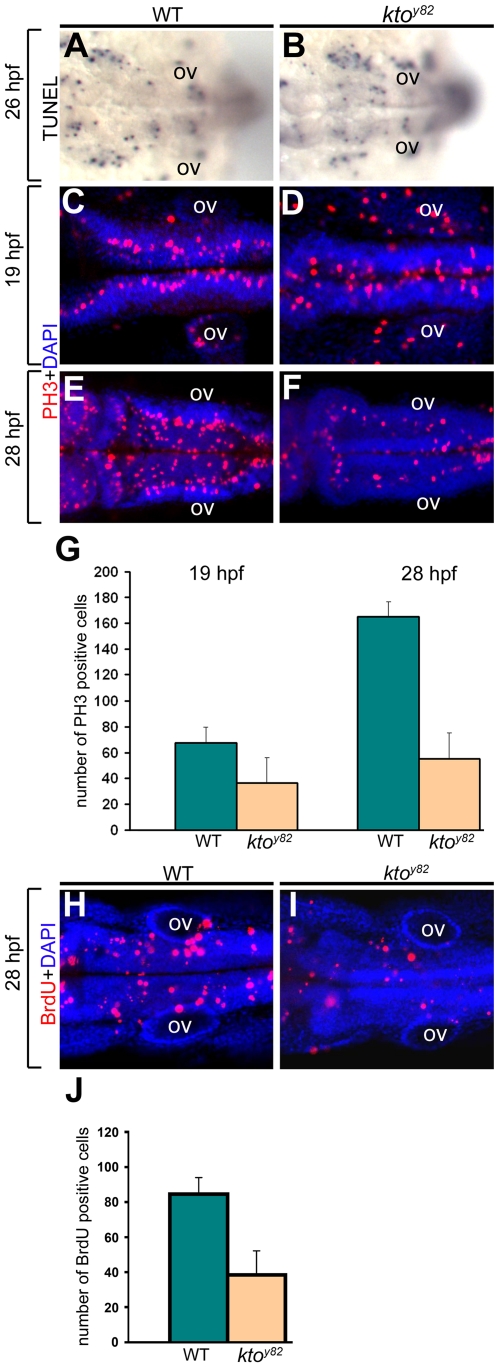
Cell proliferation defects in the hindbrain of *kto^y82^* mutants. All images are dorsal views at 19 hpf (C,D), 26 hpf (A,B), and 28 hpf (E,F,H,I). (A,B) Analysis of cell death using TUNEL assay. (C–G) PH3 staining (C–F), and quantification (G) of PH3 positive cells in wt and *kto* embryos. (H–J) BrdU staining of wt and mutant embryos (H,I), and quantification of BrdU positive cells (J). Five embryos were counted for each condition; the error bars indicate 1 standard deviation based on 5 samples. PH3 staining at 19 hpf, wt vs. *kto*: p = 0.003456; at 28 hpf, wt vs. *kto*: p = 2.97e-05. BrdU staining, wt vs. *kto*: p = 0.000706. ov, otic vesicle.

Even though the hindbrain undergoes at least some level of segmentation in *kto* embryos, boundary formation is severely defective as judged by the expression of specific marker genes. We asked whether changes in cell death or cell proliferation in mutant embryos correlate with these developmental defects. We tested for apoptotic cell death at 28 hpf by performing TUNEL assay in wt and *kto* embryos. No obvious difference was seen between wt and mutants in this assay, indicating that excess apoptotic cell death is not the cause of the disruption of hindbrain segmental organization we observe in *kto* embryos (Figure 4A,B). We used two assays to test for differences in proliferation between wt and mutant hindbrain. Immunostaining with anti-phosphorylated histone 3 (PH3) antibody, which labels mitotic cells, was carried out at 19 hpf and 28 hpf, corresponding to early and late stages of hindbrain segmentation. The number of PH3-positive cells was moderately reduced at 19 hpf and greatly reduced by 28 hpf (Figure 3C–F), and the differences between mutant and wild type were highly significant at both stages (Figure 3G and legend). The second assay for proliferation involved incorporation of BrdU. Embryos were exposed to BrdU at 19 hpf and maintained in its presence until 28 hpf when they were fixed. In agreement with PH3 staining we found that BrdU incorporation decreased by more that two-fold in *kto* mutant embryos (Figure 4H–J). Again the difference is highly significant (legend to Figure 4). Thus we conclude that proliferation of cells in the hindbrain is severely affected in embryos that carry the *kto/med12* mutation.

## Discussion

Previous studies on the *kto/med12* mutant in zebrafish showed multiple defects in tissue development in the neural crest, brain, kidney and elsewhere [6], [7], [8], [9]. More recently, a hypomorphic mutation in the mouse was reported to show neural tube closure defects, while a null mutant exhibited early lethality [10]. In this study we focused on the developmental deficits generated in the hindbrain of *kto* mutant zebrafish. We found that the hindbrain undergoes some level of segmentation, but expression of many but not all rhombomere-specific genes is reduced or abolished. Most strikingly, rhombomere boundaries are not established in the hindbrain of *kto* mutant zebrafish. During animal development boundaries are formed in somites, brain, intestinal tracts, and elsewhere. The mechanism of boundary formation has been extensible studied because loss of boundaries causes defects in downstream patterning events [42]. We observed a loss of rhombomeric boundaries in *kto* embryos, but boundary formation between somites appeared normal (Figure 1), suggesting a specific requirement for Med12 function in the hindbrain. We have not examined the state of boundaries in other tissues in *kto* mutant zebrafish.

Previous studies indicate that a loss of segmental identity during early steps of hindbrain development cause defects in boundary cell differentiation at later stages [24]. In particular, even and odd-numbered rhombomeres have distinct properties, and boundary formation may depend on the apposition of different types of segments. Rhombomeres express specific genes at early stages of their specification, and these genes can be used to identify rhombomeric segments early in their development. Using such marker genes we found that specific gene expression, and thus presumably rhombomere identity, is lost in odd-numbered segments r1, r3, and r5 starting early in development, whereas even-numbered rhombomeres r2, r4, and r6 retain specific marker expression with the exception of the loss of some markers in r6 (Figure 2). These observations suggest that the affected genes depend on Med12 function for their expression, but it is not known whether this effect is direct. If odd-numbered rhombomeres do indeed lose their identity in *kto* mutant zebrafish, the loss of boundaries in these mutants would be predicted by the hypothesis that requires the apposition of distinct segments for boundary formation.

The mechanism of formation of segmental boundaries in the hindbrain has been studied in considerable detail. Eph-ephrin signaling is a crucial component of the segmentation process, contributing to adhesion and repulsion between cells of the same or different segments, respectively [23], [24], [25], [26]. We found that *kto* mutant embryos continue to express *efnb3b* at a normal level, but *epha4a* expression was lost (Figure 2K,L,S,T). This observation by itself predicts a loss of all rhombomere boundaries with the possible exception of r6/7. Whether specified by the apposition of distinct segments or another mechanism, boundary formation involves Notch and Wnt signaling [27], [28], [29], [30], [31]. We found that components of both signaling pathways are lost in the hindbrain of *kto* mutant zebrafish. This is the case for *notch1a* and for *delta D2* (Figure 3I–L), and most strikingly for the Notch signaling amplifier *rfng* that normally marks rhombomere boundaries but is undetectable in mutant zebrafish (Figure 3C–F). Likewise *wnt1*, which is expressed at the dorsal midline and in segmental boundaries in the hindbrain, was lost in Med12 MO-injected embryos (Figure S1A,B). Thus the major signaling pathways involved in boundary formation were impaired in the absence of Med12 function in the zebrafish embryo.

The *kto/med12* mutation leads to defects in certain neuronal subtypes and in the formation of cranial sensory ganglia, while many neuronal subtypes in the central nervous system develops normally [9]. We found that boundary specific commissural axons failed to differentiate in the mutant embryo, a defect that correlatest with the close apposition of these neurons to rhombomere boundaries (Figure 3G,H).

The mechanism underlying the loss of rhombomere boundaries in *kto* mutants is not fully understood, but may involve the loss of odd-numbered segmental identity. We further explored the possibility that cell death or proliferation plays a role in this effect. We found that apoptotic cell death was not obviously increased in *kto* embryos, but cell proliferation was clearly affected. This was the case as assayed by BrdU incorporation as well as by PH3 staining at two stages of development (Figure 4). The basis for this effect is not understood at present. It is possible that it is a consequence of the loss of expression of regulatory genes during hindbrain segmentation, or there might be a direct requirement for Med12 function in proliferation or in the synthesis of components of the machinery that supports DNA replication and cell division. If the latter interpretation is correct there should be a similar reduction in proliferation in all tissues in *kto* mutant embryos, but such a general reduction in proliferation was not detected in a previous study [7]. Thus, the specific effect of Med12 deficiency on hindbrain differentiation appears to be linked to a specific deficit in cell proliferation. While the ensuing reduction in cell numbers undoubtedly contributes to the abnormal hindbrain development in *kto/med12* mutants it appears unlikely that it can explain the specific loss of rhombomere boundaries, which are more likely due to impaired segmental identity in the mutant hindbrain.

## Materials and Methods

### Zebrafish lines

Wild and kto^y82^ mutant embryos [6] were raised at 28.5°C and selection of embryos stages were according to [43]. The transgenic line pGFP5.3 [38] was kindly provided by Cecilia Moens with approval from Michael Brand. This work has been approved by the NICHD Animal Use and Care Committee under Animal Study Proposal 09–039.

### Whole-mount in situ hybridization

Digoxigenin-11-UTP or Fluorescein-12-UTP labeled probes for single or double whole-mount in situ hybridization were synthesized according to manufacturer's instructions (Roche). Whole-mount in situ hybridization [44], [45] and two-color in situ hybridization protocols [46] have been reported. We used BM Purple (Roche) for signal detection.

### Morpholino

The anti-sense oligonucleotide sequence of Med12 MO, originally named Trap230 MO, has been described previous [6]. Two ng of Trap230 and 10 ng of standard control MO were injected into one-cell stage embryos.

### Immunostaining

Monoclonal zn-5 antibody was purchased from Zebrafish International Resource Center (Eugene, OR), and zebrafish deltaD anti-body zdD2 is from Abcam. Primary anti-phospho-histone H3(PH3) polyclonal antibody (1∶500; Upstate) was used, and detected using Alexa Fluor 488-congugated anti-mouse IgG as a secondary antibody (1∶10,000; Invitrogen). Laser scanning confocal imaging was done with a Zeiss LSM 510 confocal microscope.

### TUNEL assay and BrdU incorporation

The reagents for the TUNEL assay were purchased from Invitrogen and used as described [6]. For BrdU labeling, manually dechorionated 19 hpf zebrafish embryos were placed for 30 minutes in a 8°C water bath in a solution of 10 mM BrdU in Ringer's solution (Roche) containing 15% DMSO. After 30 minutes, embryos were quickly rinsed with Ringer's solution then incubated to the desired stages before fixation.

## Supporting Information

Figure S1 **Injection of Med12 MO recapitulates the hindbrain boundary phenotype of the** ***kto*** **mutant.** All images are dorsal views of control MO (A,C,E,G) and Med12 MO (formerly called Trap230 MO) (B,D,F,H) injected embryos at 24 hpf. *Wnt1* (A,B), *rfng* (C,D), *foxb1.2* (E,F), and *notch1a* (G,H) were used as hindbrain boundary markers. ov, otic vesicle.
(PDF)

## References

[1] Conaway RC, Sato S, Tomomori-Sato C, Yao T, Conaway JW (2005). The mammalian Mediator complex and its role in transcriptional regulation.. Trends Biochem Sci.

[2] Malik S, Roeder RG (2010). The metazoan Mediator co-activator complex as an integrative hub for transcriptional regulation.. Nat Rev Genet.

[3] Malik S, Roeder RG (2005). Dynamic regulation of pol II transcription by the mammalian Mediator complex.. Trends Biochem Sci.

[4] Bjorklund S, Gustafsson CM (2005). The yeast Mediator complex and its regulation.. Trends Biochem Sci.

[5] Bourbon HM (2008). Comparative genomics supports a deep evolutionary origin for the large, four-module transcriptional mediator complex.. Nucleic Acids Res.

[6] Hong SK, Haldin CE, Lawson ND, Weinstein BM, Dawid IB (2005). The zebrafish kohtalo/trap230 gene is required for the development of the brain, neural crest, and pronephric kidney.. Proc Natl Acad Sci U S A.

[7] Shin CH, Chung WS, Hong SK, Ober EA, Verkade H (2008). Multiple roles for Med12 in vertebrate endoderm development.. Dev Biol.

[8] Rau MJ, Fischer S, Neumann CJ (2006). Zebrafish Trap230/Med12 is required as a coactivator for Sox9-dependent neural crest, cartilage and ear development.. Dev Biol.

[9] Wang X, Yang N, Uno E, Roeder RG, Guo S (2006). A subunit of the mediator complex regulates vertebrate neuronal development.. Proc Natl Acad Sci U S A.

[10] Rocha PP, Scholze M, Bleiss W, Schrewe H (2010). Med12 is essential for early mouse development and for canonical Wnt and Wnt/PCP signaling.. Development.

[11] Ding N, Zhou H, Esteve PO, Chin HG, Kim S (2008). Mediator links epigenetic silencing of neuronal gene expression with x-linked mental retardation.. Mol Cell.

[12] Philibert RA, Madan A (2007). Role of MED12 in transcription and human behavior.. Pharmacogenomics.

[13] Risheg H, Graham JM, Clark RD, Rogers RC, Opitz JM (2007). A recurrent mutation in MED12 leading to R961W causes Opitz-Kaveggia syndrome.. Nat Genet.

[14] Lumsden A, Krumlauf R (1996). Patterning the vertebrate neuraxis.. Science.

[15] Trainor PA, Krumlauf R (2000). Patterning the cranial neural crest: hindbrain segmentation and Hox gene plasticity.. Nat Rev Neurosci.

[16] Fraser S, Keynes R, Lumsden A (1990). Segmentation in the chick embryo hindbrain is defined by cell lineage restrictions.. Nature.

[17] Guthrie S, Lumsden A (1991). Formation and regeneration of rhombomere boundaries in the developing chick hindbrain.. Development.

[18] McKay IJ, Muchamore I, Krumlauf R, Maden M, Lumsden A (1994). The kreisler mouse: a hindbrain segmentation mutant that lacks two rhombomeres.. Development.

[19] Moens CB, Yan YL, Appel B, Force AG, Kimmel CB (1996). valentino: a zebrafish gene required for normal hindbrain segmentation.. Development.

[20] Lecaudey V, Anselme I, Rosa F, Schneider-Maunoury S (2004). The zebrafish Iroquois gene iro7 positions the r4/r5 boundary and controls neurogenesis in the rostral hindbrain.. Development.

[21] Maves L, Kimmel CB (2005). Dynamic and sequential patterning of the zebrafish posterior hindbrain by retinoic acid.. Dev Biol.

[22] Schneider-Maunoury S, Topilko P, Seitandou T, Levi G, Cohen-Tannoudji M (1993). Disruption of Krox-20 results in alteration of rhombomeres 3 and 5 in the developing hindbrain.. Cell.

[23] Cooke JE, Kemp HA, Moens CB (2005). EphA4 is required for cell adhesion and rhombomere-boundary formation in the zebrafish.. Curr Biol.

[24] Cooke JE, Moens CB (2002). Boundary formation in the hindbrain: Eph only it were simple.. Trends Neurosci.

[25] Kemp HA, Cooke JE, Moens CB (2009). EphA4 and EfnB2a maintain rhombomere coherence by independently regulating intercalation of progenitor cells in the zebrafish neural keel.. Dev Biol.

[26] Xu Q, Mellitzer G, Robinson V, Wilkinson DG (1999). In vivo cell sorting in complementary segmental domains mediated by Eph receptors and ephrins.. Nature.

[27] Cheng YC, Amoyel M, Qiu X, Jiang YJ, Xu Q (2004). Notch activation regulates the segregation and differentiation of rhombomere boundary cells in the zebrafish hindbrain.. Dev Cell.

[28] Qiu X, Lim CH, Ho SH, Lee KH, Jiang YJ (2009). Temporal Notch activation through Notch1a and Notch3 is required for maintaining zebrafish rhombomere boundaries.. Dev Genes Evol.

[29] Amoyel M, Cheng YC, Jiang YJ, Wilkinson DG (2005). Wnt1 regulates neurogenesis and mediates lateral inhibition of boundary cell specification in the zebrafish hindbrain.. Development.

[30] Riley BB, Chiang MY, Storch EM, Heck R, Buckles GR (2004). Rhombomere boundaries are Wnt signaling centers that regulate metameric patterning in the zebrafish hindbrain.. Dev Dyn.

[31] Blair SS (2004). Developmental biology: Notching the hindbrain.. Curr Biol.

[32] Moens CB, Cordes SP, Giorgianni MW, Barsh GS, Kimmel CB (1998). Equivalence in the genetic control of hindbrain segmentation in fish and mouse.. Development.

[33] Kelly GM, Erezyilmaz DF, Moon RT (1995). Induction of a secondary embryonic axis in zebrafish occurs following the overexpression of beta-catenin.. Mech Dev.

[34] Thisse B, Pflumio S, Fürthauer M, Loppin B, Heyer V (2001). Expression of the zebrafish genome during embryogenesis.:.

[35] McClintock JM, Kheirbek MA, Prince VE (2002). Knockdown of duplicated zebrafish hoxb1 genes reveals distinct roles in hindbrain patterning and a novel mechanism of duplicate gene retention.. Development.

[36] Hernandez RE, Putzke AP, Myers JP, Margaretha L, Moens CB (2007). Cyp26 enzymes generate the retinoic acid response pattern necessary for hindbrain development.. Development.

[37] Nittenberg R, Patel K, Joshi Y, Krumlauf R, Wilkinson DG (1997). Cell movements, neuronal organisation and gene expression in hindbrains lacking morphological boundaries.. Development.

[38] Picker A, Scholpp S, Bohli H, Takeda H, Brand M (2002). A novel positive transcriptional feedback loop in midbrain-hindbrain boundary development is revealed through analysis of the zebrafish pax2.1 promoter in transgenic lines.. Development.

[39] Sassa T, Aizawa H, Okamoto H (2007). Visualization of two distinct classes of neurons by gad2 and zic1 promoter/enhancer elements in the dorsal hindbrain of developing zebrafish reveals neuronal connectivity related to the auditory and lateral line systems.. Dev Dyn.

[40] Matsuda M, Chitnis AB (2009). Interaction with Notch determines endocytosis of specific Delta ligands in zebrafish neural tissue.. Development.

[41] Bierkamp C, Campos-Ortega JA (1993). A zebrafish homologue of the Drosophila neurogenic gene Notch and its pattern of transcription during early embryogenesis.. Mech Dev.

[42] Dahmann C, Oates AC, Brand M (2011). Boundary formation and maintenance in tissue development.. Nat Rev Genet.

[43] Kimmel CB, Ballard WW, Kimmel SR, Ullmann B, Schilling TF (1995). Stages of embryonic development of the zebrafish.. Dev Dyn.

[44] Kudoh T, Tsang M, Hukriede NA, Chen X, Dedekian M (2001). A gene expression screen in zebrafish embryogenesis.. Genome Res.

[45] Toyama R, O'Connell ML, Wright CV, Kuehn MR, Dawid IB (1995). Nodal induces ectopic goosecoid and lim1 expression and axis duplication in zebrafish.. Development.

[46] Hauptmann G, Gerster T (1994). Two-color whole-mount in situ hybridization to vertebrate and Drosophila embryos.. Trends Genet.

