# Supplementary figures and images for: The Transcriptional Mediator Component Med12 Is Required for Hindbrain Boundary Formation
Source: PLoS One. 2011 Apr 21;6(4):e19076. doi: 10.1371/journal.pone.0019076 (PMC3080914; doi:10.1371/journal.pone.0019076)

Ctrl MO

Trap230 MO

*wnt1*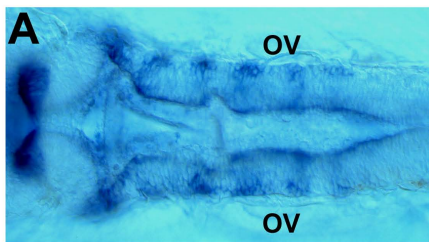**B**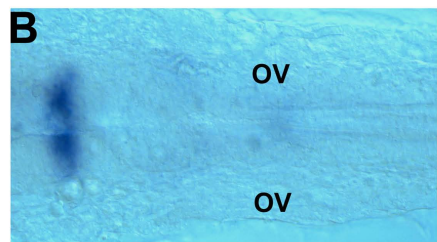*rfng*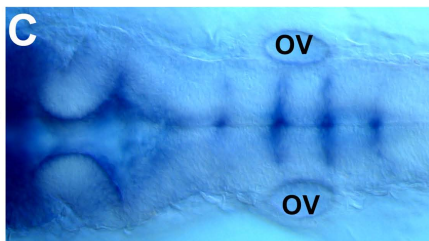**D**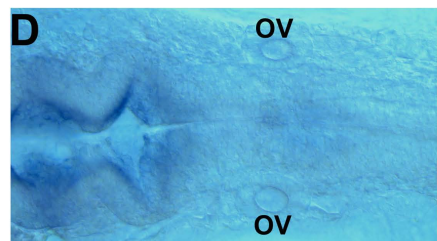*foxb1.2*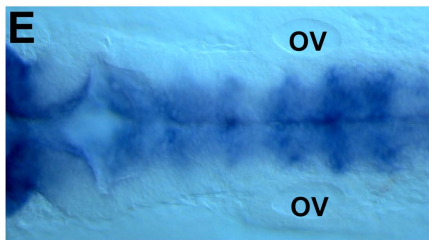**F**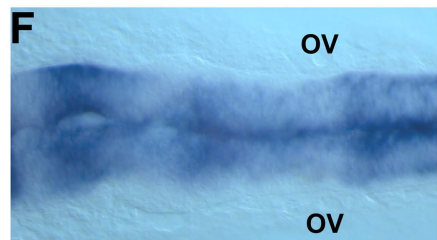*notch1a*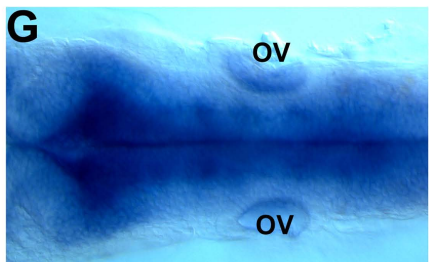**H**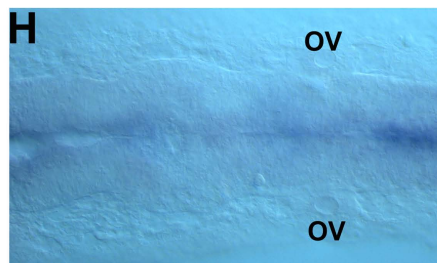

Supplement: Figure S1 — Injection of Med12 MO recapitulates the hindbrain boundary phenotype of the kto mutant. All images are dorsal views of control MO (A,C,E,G) and Med12 MO (formerly called Trap230 MO) (B,D,F,H) injected embryos at 24 hpf. Wnt1 (A,B), rfng (C,D), foxb1.2 (E,F), and notch1a (G,H) were used as hindbrain boundary markers. ov, otic vesicle. (PDF) [file pone.0019076.s001.pdf]
